# Empagliflozin’s role in reducing ventricular repolarization heterogeneity: insights into cardiovascular mortality decline from the EMPATHY-HEART trial

**DOI:** 10.1186/s12933-024-02311-1

**Published:** 2024-06-26

**Authors:** Cristiane Lauretti, Graziella L. Antonio, Ariana E. Fernandes, Fernando G. Stocco, Adriana C. C. Girardi, Richard L. Verrier, Bruno Caramelli

**Affiliations:** 1 Interdisciplinary Medicine Unit in Cardiology, Heart Institute of the Clinical Hospital of the Medical School of the University of Sao Paulo, Av. Dr. Enéas Carvalho de Aguiar, 44- Anexo II, Sao Paulo, 05403000 SP Brazil; 2 Medical School Laboratory of Genetics and Molecular Cardiology , Heart Institute of the Clinical Hospital University of Sao Paulo , Sao Paulo, 05403000 Brazil SP; 3 Harvard Medical School and Beth Israel Deaconess Medical Center, Boston, MA 02215 United States of America

**Keywords:** Type 2 diabetes Mellitus, Coronary heart disease, Empagliflozin, T-wave heterogeneity, Ventricular Arrhythmias, Cardiovascular mortality, Non-invasive diagnostic tools, EMPA-REG Trial, Electrical instability, Arrhythmia prevention

## Abstract

**Background:**

The incidence of myocardial infarction (MI) and sudden cardiac death (SCD) is significantly higher in individuals with Type 2 Diabetes Mellitus (T2DM) than in the general population. Strategies for the prevention of fatal arrhythmias are often insufficient, highlighting the need for additional non-invasive diagnostic tools. The T-wave heterogeneity (TWH) index measures variations in ventricular repolarization and has emerged as a promising predictor for severe ventricular arrhythmias. Although the EMPA-REG trial reported reduced cardiovascular mortality with empagliflozin, the underlying mechanisms remain unclear. This study investigates the potential of empagliflozin in mitigating cardiac electrical instability in patients with T2DM and coronary heart disease (CHD) by examining changes in TWH.

**Methods:**

Participants were adult outpatients with T2DM and CHD who exhibited TWH > 80 µV at baseline. They received a 25 mg daily dose of empagliflozin and were evaluated clinically including electrocardiogram (ECG) measurements at baseline and after 4 weeks. TWH was computed from leads V4, V5, and V6 using a validated technique. The primary study outcome was a significant (*p* < 0.05) change in TWH following empagliflozin administration.

**Results:**

An initial review of 6,000 medical records pinpointed 800 patients for TWH evaluation. Of these, 412 exhibited TWH above 80 µV, with 97 completing clinical assessments and 90 meeting the criteria for high cardiovascular risk enrollment. Empagliflozin adherence exceeded 80%, resulting in notable reductions in blood pressure without affecting heart rate. Side effects were generally mild, with 13.3% experiencing Level 1 hypoglycemia, alongside infrequent urinary and genital infections. The treatment consistently reduced mean TWH from 116 to 103 µV (*p* = 0.01).

**Conclusions:**

The EMPATHY-HEART trial preliminarily suggests that empagliflozin decreases heterogeneity in ventricular repolarization among patients with T2DM and CHD. This reduction in TWH may provide insight into the mechanism behind the decreased cardiovascular mortality observed in previous trials, potentially offering a therapeutic pathway to mitigate the risk of severe arrhythmias in this population.

**Trial registration:**

NCT: 04117763.

## Background

The global incidence of Type 2 Diabetes Mellitus (T2DM) is reaching critical levels, projected to affect approximately 600 million individuals by 2035 [1]. This condition markedly increases the prevalence of cardiovascular diseases (CVD) and generates a concern highlighted by the higher rates of post-myocardial infarction (MI) mortality and a greater frequency of sudden cardiac death (SCD) among those with diabetes, in comparison to non-diabetic individuals [2, 3].

Often, SCD is the first clinical indication of an underlying, undetected cardiac disorder. Traditional risk factors fall short in accurately predicting these fatal arrhythmias, underscoring the need for more precise predictive tools [4]. SCD is generally preceded by an electrical disturbance, prompting recent shifts towards non-invasive methods for risk assessment using electrocardiographic indicators [5]. One such marker is the T-wave heterogeneity index (TWH) that measures the variance of waveforms around the T-wave’s average waveform, effectively capturing the spatial disparity in heart repolarization [4]. TWH has proven valuable in accurately stratifying the risk of sudden cardiac death, overall cardiac mortality, and arrhythmic episodes in a diverse range of cardiovascular conditions [6–9].

Empagliflozin, a sodium-glucose cotransporter-2 inhibitor (SGLT2i), has emerged as a key player in cardiovascular healthcare, a status underscored by EMPA-REG trial [10]. This study highlighted a 14% reduction in relative risk for a combined outcome of cardiovascular death, non-fatal MI, and non-fatal stroke, with a notable 38% decrease in cardiovascular mortality, including sudden death cases. These benefits were significant even in patients already under comprehensive cardiovascular risk management and were observable as soon as 27 days post-treatment initiation [11]. The rapid and significant effects of empagliflozin on cardiovascular outcomes in patients with high-risk T2DM are acknowledged, yet the mechanisms behind these effects remain partly unclear. Dapagliflozin, another SGLT2i, has been shown to reduce ventricular arrhythmias, cardiac arrests, and SCD in conjunction with standard heart failure (HF) treatments [12]. Additionally, preclinical studies bolster the hypothesis that empagliflozin may diminish the risk of fatal arrhythmias [13]. These findings are crucial for managing patients with high-risk cardiovascular diabetes and underscore the need for focused clinical trials to investigate further the potential effect of SGLT2i in the prevention of cardiac arrhythmias. While there’s evidence suggesting a potential reduction in fatal arrhythmias with empagliflozin [14], its specific anti-arrhythmic actions and influence on SCD susceptibility in T2DM and CHD patients still require further elucidation. Our study aimed to explore whether empagliflozin can attenuate ventricular arrhythmogenesis and lower the risk of SCD by assessing TWH in diabetic patients with coronary artery disease, contributing to the evolving field of cardiovascular care in patients with diabetes.

## Methods

### Study design

The EMPATHY-HEART Trial employed an exploratory pilot study design to investigate the influence of empagliflozin on ventricular repolarization heterogeneity in patients diagnosed with both T2DM and CHD. The choice of an exploratory design emerged from the imperative to elucidate the potential impacts of empagliflozin on ventricular electrical instability, an aspect with limited prior exploration. Given the limited number of clinical trials utilizing interlead T-wave heterogeneity, the study aimed to establish the basis for future research and hypothesis development.

### Participants

Outpatients at Heart Institute of the Faculty of Medicine of the University of Sao Paulo (InCor), Brazil, were invited to participate. The study included individuals 18 years and older, diagnosed with T2DM, and showing evidence of CHD, indicated by a history of myocardial infarction, significant coronary stenosis, or a positive test for myocardial ischemia. Additionally, participants required a baseline TWH on ECG of 80 µV or higher, following the criteria established by Tan et al. [7]. Exclusion criteria comprised chronic kidney disease (glomerular filtration rate below 45 ml/min/1.73 m²); advanced hepatic disease (Child-Pugh B or C); age over 85 years and an uninterpretable 12-lead baseline ECG, due to conditions like pacemaker rhythms or signal distortions. In this context, “baseline” ECG refers to the electrocardiogram conducted at the first study visit.

### Intervention

Under the guidance of a sole researcher (CL), all participants underwent comprehensive clinical assessments. They received a daily prescription of empagliflozin 25 mg, aligning with current clinical guidelines emphasizing its cardiovascular benefits in high-risk diabetic populations. This dosage selection aimed specifically to investigate empagliflozin’s effects on the prevention of cardiac arrhythmias. Tailored adjustments to hypoglycemic regimens were made as necessary to minimize the risk of hypoglycemia, ensuring a standardized yet personalized approach for participant safety and treatment efficacy.

Patients performed a 12-lead ECG at their initial visit and again after 4 weeks. During the follow-up, a clinical reassessment and treatment adherence analysis were conducted. This 4-week period aligns with findings from a post-hoc analysis indicating a notable decrease in cardiovascular death or HF hospitalization starting from day 27 post-randomization [11]. Treatment adherence was evaluated through direct questioning of each participant [15].

### Data collection

Data Management and Statistical Analysis Data acquisition and management were conducted using the REDCap (Research Electronic Data Capture) platform [16].

Resting electrocardiograms were conducted using Mortara Eli 250 C (2013) or GE Healthcare MAC 2000 (2019) equipment, capturing 12 leads simultaneously in a 4 × 3 format, with a paper speed of 25 mm/s and a 10-mV gain. Electrode placement followed standard technical guidelines [17].

TWH was calculated specifically in leads V4, V5, and V6, chosen for their lower variability due to electrode positioning and patient’s body constitution [18], and their proven effectiveness in detecting electrical instability and arrhythmia risk [6, 7].

Two independent researchers, CL and GLA, blinded to the patients’ treatment status, performed the TWH calculations for each ECG.

Participant ECGs were extracted from InCor’s electronic records as PDFs, anonymized, and converted to TIFF format. ECGScan software (AMPS-LLC, New York, NY) translated the waveforms [19], which were then turned into text files using ISHNE and CalECG softwares (AMPS-LLC, New York, NY) [20]. These files were analyzed in Microsoft Excel (365 version). Data from leads V4, V5, and V6 were overlaid, aligned by the PR segment, and synchronized at the QRS complex onset. The second central moment algorithm was applied to the J-T wave interval of each cardiac cycle to determine the point of highest morphology variance. The square root of this variance provided the TWH value in microvolts per beat. The TWH index, representing the average TWH of all recorded beats, indicates repolarization nonuniformity; higher values suggest a greater risk of life-threatening ventricular arrhythmias [7]. Detailed TWH calculation methods from resting 12-lead ECGs are described elsewhere [4, 5, 9].

### Statistical analysis

The primary goal of our study was to investigate how TWH levels were altered by empagliflozin administration. To ensure reliability and agreement of ECG data analyzed by the independent researchers we utilized the Intraclass Correlation Coefficient (ICC). To enhance result precision, we excluded extreme outliers, namely, subjects whose TWH levels were more than 3 standard deviations above the population mean, to reduce data skewness. The Shapiro-Wilk test was employed for normality evaluation. Variables were analyzed using Student’s t-test for normal distributions and the Wilcoxon rank-sum test for non-parametric data, including TWH, to determine empagliflozin’s effects. Multivariate analysis utilized MANOVA, with all tests adhering to a significance threshold of *p* < 0.05, reflecting a 5% significance level. Statistical analyses were conducted using R, a software environment for statistical computing and graphics [21]. Data are presented as means ± standard deviation (S.D.)

### Ethical considerations

Recruitment for this study occurred between October 2019 and October 2023. Each participant gave written informed consent, having been fully briefed on aims, methods, and potential risks. Adhering to the Declaration of Helsinki’s principles, this clinical trial was approved by the Institutional Review Board of Clinical Hospital of the Medical School of the University of Sao Paulo, under registration number SDC: 4732/18/083. The research is registered on ClinicalTrials.gov with the identifier NCT04117763.

## Results

### Patient characteristics

**Fig. 1:**
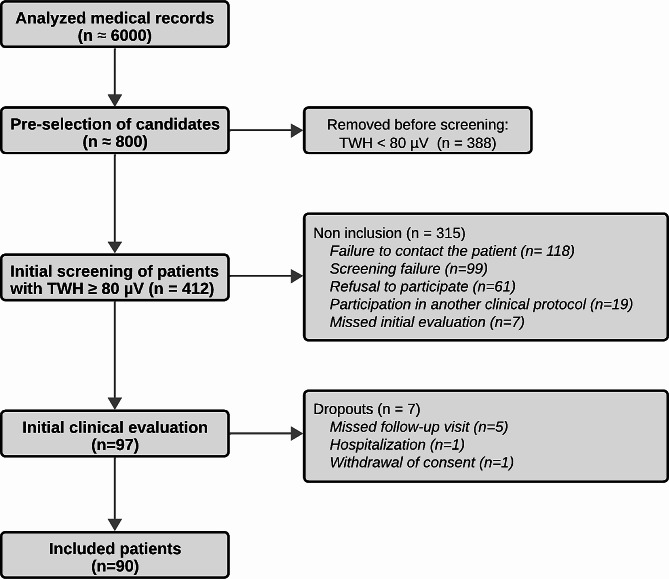
Participant inclusion flowchart

 Among the potential candidates screened, 412 had TWH equal to or greater than 80 µV. Of these, 90 participants were included in the study. Figure 1 illustrates the flowchart of participant inclusion. The demographic and clinical profiles of the participants, as outlined in Table 1, indicated a cohort with a notably high cardiovascular risk. This included the majority having experienced myocardial infarctions, over half displaying a tri-arterial pattern in invasive stratification, and nearly a quarter having undergone coronary artery bypass grafting. Only one patient was included based on the criterion of a positive non-invasive test for myocardial ischemia. As expected in a specialized heart hospital setting, participants were optimized for cardiovascular risk reduction. The majority were on statins (90%) and beta-blockers (84%). Angiotensin-converting enzyme inhibitors or angiotensin receptor blockers were used by 77% of the patients. Additionally, 11% were on furosemide, 21% on spironolactone, and none on sacubitril/valsartan. No modifications were made to the HF treatment regimen of the patients, except for the addition of empagliflozin. For diabetes management, 24% were on basal insulin therapy, and 11% adhered to a basal-bolus insulin therapy regimen. Only two subjects were not on antiplatelet agents.

**Table 1:** Patient characteristics

Characteristic	
Sex: Male	89%
Age: Mean (years)	64 ± 7.5
Race: White	80%
*DM2 Duration* ≤ 10 years > 10 years	46% 54%
*Glycemic control* HbA1c < 8.0 HbA1c ≥ 8.0	69% 31%
Hypertension	96%
Obesity	26%
Current smoking	10%
Previous stroke	7%
Peripheral arterial disease	12%
Previous Myocardial infarction	87%
Invasive stratification Uniarterial Biarterial Triarterial	98% 15% 26% 58%
Angina	48%
Percutaneous Revascularization	61%
Surgical Revascularization	24%
*Heart Failure* Preserved EF Reduced EF	82% 61% 39%
Dyslipidemia	100%
*Lipid Control (med, mg/dL)*	
LDL	78 ± 23
HDL	40 ± 11
Triglycerides	134 ± 71

### Clinical parameters

Adherence to treatment exceeded 80% across all participants. Consistent with prior evidence [10], our study found that empagliflozin significantly reduced blood pressure without significantly affecting heart rate (Table 2).

**Table 2:** Clinical Characteristics of the Participants

Measurements	Baseline (mean)	Follow-up (mean)	*p*-value
Systolic BP (mmHg)	137.0	126.0	0.0002
Diastolic BP (mmHg)	77.6	72.6	0.009
Heart Rate (bpm)	71.9	71.7	0.96
Weight (kg)	77.8	77.1	0.70

Hypoglycemia was noted in 13.3% of participants, with a predominant occurrence at Level 1 [15] (91.7%), and no reports of serious episodes. Urinary tract infections were observed in 4.4%, while genital fungal infections occurred in 11.1% of the patients.

### T-Wave heterogeneity

TWH values for each ECG, assessed by independent researchers, showed high reliability, as reflected by ICC values of 0.9 for both baseline and follow-up phases, with p-values < 0.001. This robust concordance validated aggregating data from both researchers for subsequent data analysis and presentation.

Following empagliflozin treatment, we noted a significant reduction in TWH values, indicating a decrease in ventricular repolarization heterogeneity. Specifically, mean baseline TWH values fell from 127 µV to 114 µV at follow-up, with a median reduction from 116 µV to 103 µV. This change, statistically significant with a p-value of 0.01, underscores a meaningful difference attributed to empagliflozin treatment.

**Fig. 2:**
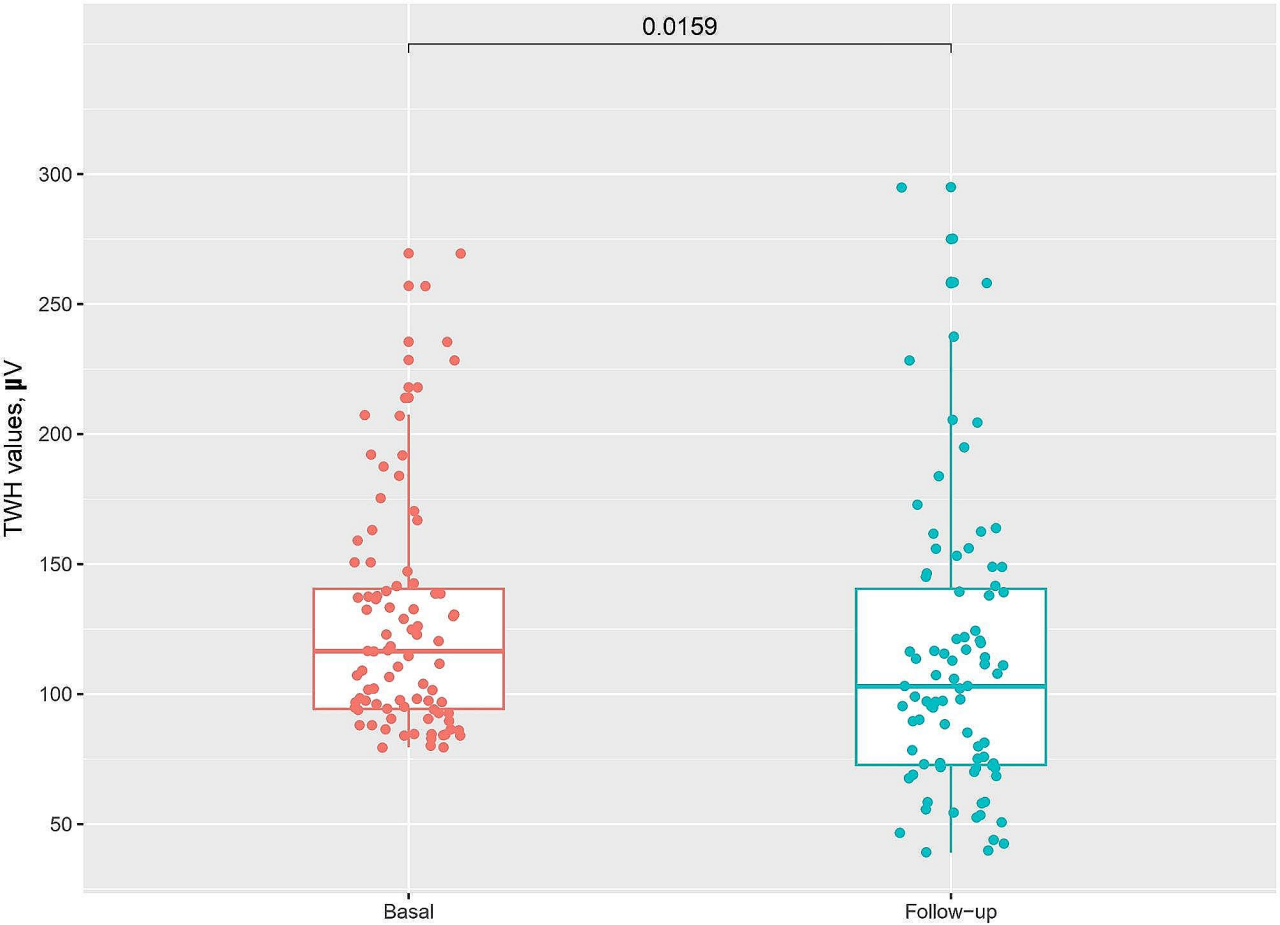
Boxplots of TWH levels in patients before (left) and after (right) empagliflozin treatment over 4 weeks

Visual analysis of the data presented in the Boxplot in Fig. 2 indicates increased variability in TWH values post-treatment, as shown by the rise in standard deviation from 43.9 to 55.7 µV and in the interquartile range from 46.2 to 67.8 µV. The presence of outliers above the third quartile, both before and after treatment, highlights a persistent dispersion in TWH values, irrespective of therapy.

**Fig. 3:**
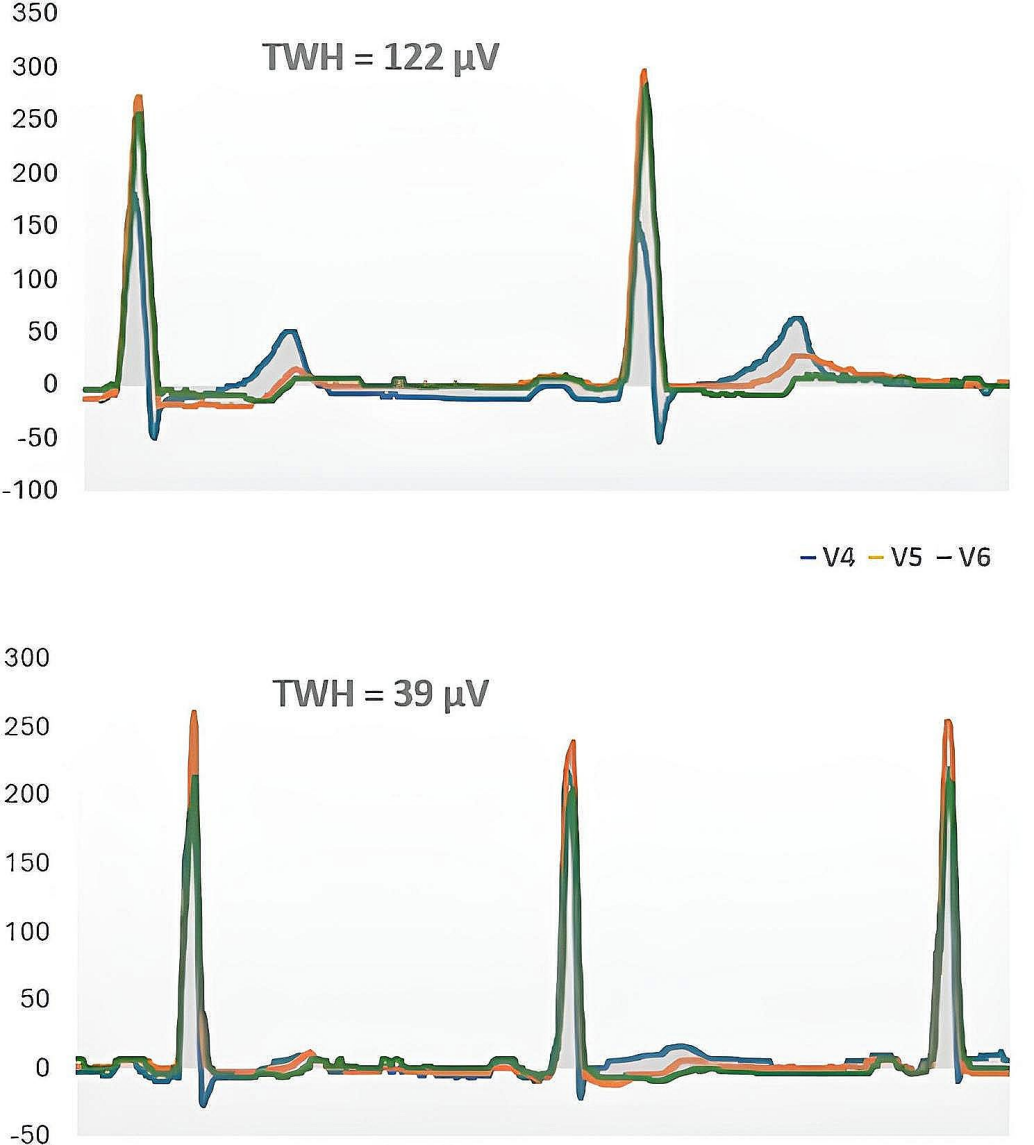
Digitized ECG tracings illustrate T- wave heterogeneity (TWH) as interlead splay in repolarization morphology before (upper panel) and after empagliflozin for 4 weeks (lower panel) in a representative participant

Representative digitized ECG tracings for a patient during the study are provided in Fig. 3. The baseline ECG, depicted in the upper panel, shows pronounced T-wave heterogeneity, with significant variability in T-wave morphology across different leads. Following a 4-week treatment with empagliflozin, the lower panel presents ECG tracings that demonstrate a noticeable reduction in TWH, evidenced by a decrease in variability among T-wave forms.

**Fig. 4:**
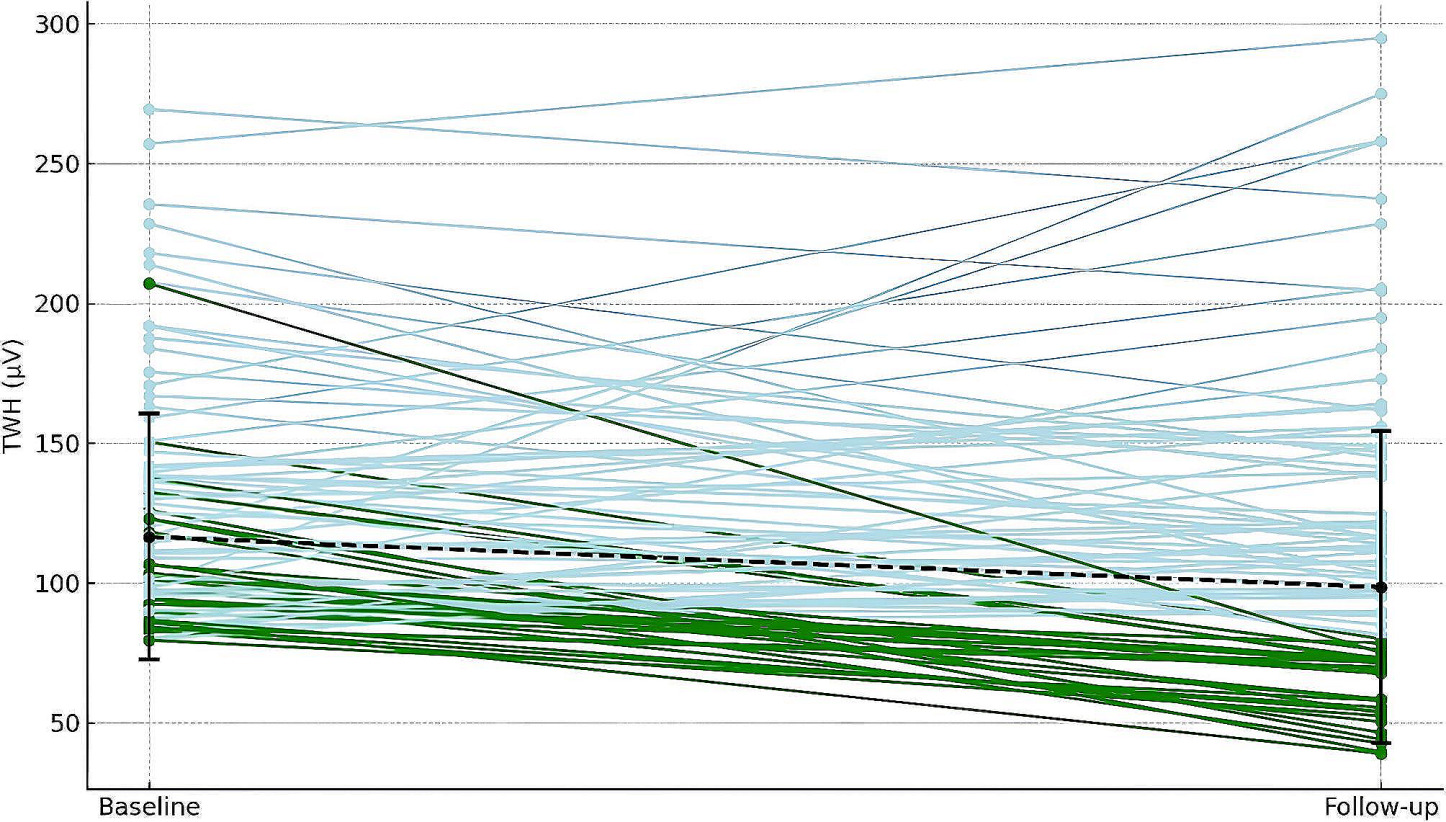
TWH before and after 4 weeks of empagliflozin (25 mg daily). Green lines: patients with TWH ≤ 80 µV post-treatment. Dashed lines denote medians before and after treatment. Significant median reduction (*p* = 0.0159)

Figure 4 reveals a trend toward lower TWH levels after treatment across the study population. About one-third of the participants achieved a TWH reduction to 80 µV or below post-treatment. Predominantly, the optimal responders – those whose TWH diminished to 80 uV or less – initially had TWH values below 100 uV.

A multivariate analytical method (MANOVA) was employed to explore the connections among clinical variables, including HbA1c levels, time since diabetes diagnosis, incidence of angina, previous MI, MI localization, classification in invasive stratification, and Left Ventricular Ejection Fraction (LVEF), with the goal of identifying a significant association with TWH reduction. In this analysis, LVEF was categorized into two groups: patients with an LVEF greater than or equal to 50%, representing preserved ejection fraction, and those with an LVEF below 50%, representing reduced ejection fraction. HbA1c levels were similarly categorized with a cutoff at 8%. Single-vessel disease and diabetes duration emerged as significant factors, leading to their inclusion in a refined linear multivariate analysis model, which revealed that only single-vessel disease had a significant effect on reducing TWH (*p* = 0.027). Inspecting this group of patients with single-vessel disease, it was observed that the majority had a prior myocardial infarction, with the anterior wall being the most affected. The model, with a multiple R^2^ of 0.321 and an adjusted R^2^ of 0.2791, alongside a p-value of 6.183e-06, reveals moderate explanatory power and significant statistical validity.

## Discussion

Participants characterized by high cardiovascular risk and treated at a specialized cardiology hospital, had increased baseline TWH, indicating a heightened susceptibility to arrhythmias or sudden cardiac death. Therapeutically, these patients were following optimal regimens, marked by widespread use of beta-blockers. Consequently, the positive effect of empagliflozin observed in this cohort point to a possible additional therapeutic benefit in addition to beta-blockers. This finding raises the intriguing possibility that beta-blockers might have slightly concealed an even greater potential of empagliflozin to diminish ventricular electrical instability. We employed the TWH level of 80 µV established by previous research to demonstrate that the risk of severe arrhythmias and SCD increases significantly when TWH levels exceed this level [7, 8]. Electrocardiograms were performed in an ambulatory setting without provocative testing for ischemia or exertion. Within a relatively brief period of four weeks of empagliflozin treatment, there was a statistically significant reduction in the TWH median.

This proof-of-concept trial showed that empagliflozin significantly reduces ventricular electrical vulnerability in patients with T2DM and CHD, evidenced by reduced T-wave heterogeneity. The findings complement recent evidence, offering new insights into these phenomena and their implications in a clinical setting for the first time.

Research into the role of SGLT2i in arrhythmia prevention is rapidly evolving. Recent studies highlight SGLT2i’s potential to reduce arrhythmia risk and SCD in diabetic and non-diabetic individuals. Key findings include the EMBODY trial’s demonstration of empagliflozin’s improvement in heart rate variability [22], and studies from Taiwan [23] and the SGLT2-I AMI PROTECT [24] indicating a 17% reduction in new arrhythmias and fewer severe arrhythmic events among SGLT2i users, respectively.

Our research corroborates a retrospective analysis of 46 T2DM patients, showing that SGLT2i decreases QTc dispersion on 12-lead ECGs without affecting heart rate, QTc interval, or Tpeak–Tend interval, particularly in those with elevated baseline QTc dispersion [25]. While not associated with HbA1c changes, a relationship was observed with systolic blood pressure variations, and post-treatment serum electrolyte levels were stable. The findings suggest that SGLT2i improves disparity in ventricular recovery times, irrespective of its effects on blood sugar levels [26]. This effect underscores the direct cardioprotective actions of SGLT2i, supported by evidence that glycemic control alone does not significantly affect QT and Tpeak-Tend dispersion [27].

While the exact mechanisms by which SGLT2i reduces arrhythmias are under investigation, it is believed that they involve multiple processes underlying cardiac arrhythmogenesis. Interest areas include their impact on cardiovascular autonomic function. Studies have shown SGLT2i decreases sympathetic activity and increases parasympathetic activity, improving autonomic balance [22]. Their influence on specific ionic currents in cardiomyocytes, reducing late-INa and spontaneous calcium transients similarly to ranolazine and lidocaine [28], has also been explored. It is relevant that blockade of sympathetic activity [29], increasing cardiac vagal tone [30], and blockade of late-INa current [31] have been shown to reduce T-wave heterogeneity. Experimental studies suggest empagliflozin directly inhibits the NHE1 exchanger and SGLT1 in cardiomyocytes [32], reducing sodium content, improving mitochondrial function, and decreasing oxidative stress, which are additional mechanisms proposed for arrhythmia reduction. Regarding ischemia-reperfusion-related arrhythmias, experimental studies on non-diabetic rats showed empagliflozin significantly reduced ventricular arrhythmias, including ventricular tachycardia and fibrillation, and eliminated SCD vulnerability [13]. Control groups had a 69.2% mortality rate, whereas empagliflozin-treated groups recorded no sudden cardiac death, indicating empagliflozin’s cardioprotective capacity through ERK1/2 phosphorylation pathway activation. Similarly, in rabbit models, empagliflozin reduced ventricular arrhythmias by improving calcium cycling and mitochondrial function [33]. Pre-ischemic use of dapagliflozin significantly reduced infarct size and cardiomyocyte apoptosis, underscoring its cardioprotective ability and potential value in minimizing cardiac damage and enhancing post-injury cardiac function [34].

In our hypothesis-generating study, we employed the TWH index as a surrogate marker for arrhythmia and SCD, given the well-established efficacy of this marker [6, 7, 9]. The significance of TWH rests on three fundamental pillars: its capacity to stratify risk by identifying individuals with high susceptibility to adverse cardiac events; its effectiveness in predicting responses to therapeutic interventions, exemplified by cardiac resynchronization therapy; and its capability in monitoring the progression of cardiac electrical instability, aiding in the evaluation of treatment efficacy [9].

Following treatment, slightly over one-third of participants experienced a reduction in TWH to below the safety threshold of 80 µV, primarily among those with initially TWH levels around 100 µV. This finding could suggest that individuals with lower baseline TWH are more likely to experience significant treatment benefits, aligning with safer TWH levels post-treatment.

However, patients’ responses to the treatment varied, as indicated by the wide distribution and high standard deviation of observed changes. This variation could be clinically significant, suggesting that while there is a general trend towards TWH reduction, individual reactions to the treatment can vary significantly. Surprisingly, a few patients experienced an increase in TWH after the intervention. This finding, emerging from this pilot study, signals a complexity that exceeds the initial scope of the research but warrants future investigation.

Recent clinical trials have highlighted the efficacy of empagliflozin in reducing the risk of hospitalization and mortality due to HF. This effect has been observed in patients with both reduced ejection fraction (HFrEF) and preserved ejection fraction (HFpEF), and extends to individuals with or without diabetes.The EMPEROR-Reduced study [35] found that empagliflozin significantly decreased the risk of the primary composite outcome of cardiovascular death or hospitalization for worsening HF in patients with HFrEF by 25%. The EMPEROR-Preserved study [36] identified a 21% reduction in the risk of cardiovascular death or hospitalization for HF in patients with HFpEF, primarily due to a 29% reduction in HF hospitalizations. Both EMPEROR studies focused on evaluating the effects of empagliflozin in HF patients, regardless of the specific cause of their HF. In addition, it’s important to note that EMPEROR trials addressed HF as syndrome and no mechanistic insights were provided. In our study population, the prevalence of HF was notable, with 74 of 90 patients (82%) diagnosed with HF. In addition, it’s important to note that EMPEROR study addressed HF as syndrome and no mechanistic insights were provided. The etiology of HF in our patients was ischemic cardiomyopathy. For 7 patients, there was no information on left ventricular ejection fraction (LVEF); 50 patients had an LVEF above 50%, 13 had an LVEF below 40%, and 14 had an LVEF between 40% and 49%. The main contribution of our study is to provide a possible mechanism to explain the benefits of empagliflozin. Indeed, we hypothesized that the significant changes observed in TWH after empagliflozin treatment, the main finding of our study, are either exclusively or partially related to HF. However, this hypothesis requires further investigation in future studies.

Our study faces certain limitations, including a small sample size, limited duration, and a non-randomized design, which may affect the generalizability of our results. Additionally, the particular demographics of our patient cohort call for careful consideration when applying these findings to wider populations. To substantiate and broaden our conclusions, future research should involve larger, more varied groups and longer observation times.

The exact molecular mechanisms by which empagliflozin attenuates ventricular arrhythmogenesis remain to be fully elucidated. This gap highlights an opportunity for future research, particularly at the molecular and cellular levels, to unravel the intricate pathways involved in the antiarrhythmic effects of SGLT2 inhibitors.

Our findings add a piece to the complex puzzle of cardiovascular management in diabetes mellitus, underscoring empagliflozin’s potential in mitigating arrhythmic risks. While enhancing our understanding of empagliflozin’s cardiovascular benefits, our study paves the way for novel research directions and clinical applications, promising significant advancements in patient care.

## Conclusions

This pilot study demonstrated that empagliflozin reduces ventricular repolarization heterogeneity in patients with T2D and CAD, suggesting that a reduction in severe arrhythmias may be among the mechanisms contributing to the observed decrease in cardiovascular mortality with this treatment.

Exploring TWH in diabetic patients with coronary artery disease opens new avenues for standardizing treatment and simplifying methodologies across this patient population. By delving into this area, we can lay the groundwork for developing products that directly calculate TWH. This approach not only has the potential to enhance clinical outcomes but also offers a basis for innovation in patient management and treatment optimization. Such advancements could significantly contribute to individualized medicine, ensuring that interventions are more accurately tailored to individual patient profiles, thereby improving efficacy and patient care in the field of coronary and diabetic health.

## Data Availability

Data is available upon request.

## Abbreviations

CHD: Coronary Heart Disease
ECG: Electrocardiogram
HF: Heart Failure
ICC: Intraclass Correlation Coefficient
InCor: Heart Institute of the Faculty of Medicine of the University of Sao Paulo
Late-INa: Late Sodium Current
LVEF: Left Ventricular Ejection Fraction
MANOVA: Multivariate Analysis of Variance
MI: Myocardial Infarction
PDF: Portable Document Format
REDCap: Research Electronic Data Capture
SCD: Sudden Cardiac Death
SD: Standard Deviation
SGLT2i: Sodium-glucose Cotransporter-2 Inhibitor
T2DM: Type 2 Diabetes Mellitus
TIFF: Tagged Image File Format
TWH: T-Wave Heterogeneity
